# Supplementary material for: N,N-Bis(2,4-Dibenzhydryl-6-cycloalkylphenyl)butane-2,3-diimine–Nickel Complexes as Tunable and Effective Catalysts for High-Molecular-Weight PE Elastomers
Source: Molecules. 2023 Jun 19;28(12):4852. doi: 10.3390/molecules28124852 (PMC10302251; doi:10.3390/molecules28124852)
Supplement: Supplementary file 1 [file molecules-28-04852-s001.zip › molecules-2446894-supplementary.pdf]

Supporting Information for

N,N-bis(2,4-Dibenzhydryl-6-cycloalkylphenyl)butane-2,3-diimine-Nickel Complexes as Tunable and Effective Catalysts for High Molecular Weight PE Elastomers

Shu Jiang [1,2], Yuting Zheng [1,2], Irina V. Oleynik [3], Zhixin Yu [1,*], Gregory A. Solan [2,4,*], Ivan I. Oleynik [3,*], Ming Liu [2], Yanping Ma [2,4], Tongling Liang [2] and Wen-Hua Sun [2,*]

[1] School of Pharmaceutical Sciences, Changchun University of Chinese Medicine, Changchun 130117, China

[2] Key Laboratory of Engineering Plastics and Beijing National Laboratory for Molecular Sciences, Institute of Chemistry, Chinese Academy of Sciences, Beijing 100190, China

[3] Department of Chemistry, University of Leicester, University Road, Leicester LE1 7RH, UK

[4] Vorozhtsov Novosibirsk Institute of Organic Chemistry, Pr. Lavrentjeva 9, Novosibirsk 630090, Russia

* Correspondence: yuzx01@ccucm.edu.cn (Z.Y.); gas8@leicester.ac.uk, Tel.: +44-(0)116-252209 (G.A.S); oleynik@nioch.nsc.ru (I.I.O.); whsun@iccas.ac.cn, Tel.: +86-10-6255-7955 (W.-H.S.)

Table of the Contents		Page
1.	Crystallographic parameters for **Ni2** and **Ni4**	S1
2.	GPC traces of the polyethylenes	S2-S7
3.	DSC thermograms of $PE30_{E\|\mathbf{Ni2}}$, $PE30_{D\|\mathbf{Ni2}}$, $PE30_{M\|\mathbf{Ni2}}$, $PE60_{E\|\mathbf{Ni2}}$, $PE60_{D\|\mathbf{Ni2}}$ and $PE60_{M\|\mathbf{Ni2}}$	S8-S9

1. Crystallographic parameters for Ni2 and Ni4

Table S1 Crystal data and structure refinement for **Ni2** and **Ni4**

Complex	**Ni2**	**Ni4**
CCDC No.	2262894	2262895
Empirical formula	$C_{80}H_{76}Br_2N_2Ni$	$2(C_{92}H_{100}Br_2N_2Ni)$
Formula weight	1283.95	1452.27
Temperature/K	170.01(10)	169.99(12)
Crystal system	monoclinic	monoclinic
Space group	$P2_1/n$	$P2_1/c$
a/Å	12.26160(10)	25.6842(5)
b/Å	36.9305(2)	21.1531(3)
c/Å	17.5933(2)	16.2046(3)
α/°	90	90
β/°	110.3200(10)	92.606(2)
γ/°	90	90
Volume/Å^3	7470.93(12)	8794.9(3)
Z	4	2
ρ_{calc} g/cm^3	1.142	1.097
μ/mm^{-1}	1.925	1.685

F (000)	2672.0	3056.0
Crystal size/mm^3	0.3 × 0.25 × 0.15	0.18 × 0.13 × 0.1
Radiation	Cu Kα (λ = 1.54184)	Cu Kα (λ = 1.54184)
2Θ range (°)	4.786 to 155.078	6.876 to 154.142
Index ranges	$-15 \le h \le 15$, $-39 \le k \le 46$, $-20 \le l \le 22$	$-32 \le h \le 30$, $-26 \le k \le 26$, $-14 \le l \le 20$
No. of rflns collected	50978	17651
No. unique rflns (R_{int})	14863 [R_{int} = 0.0217, R_{sigma} = 0.0201]	17651 [R_{int} = ?, R_{sigma} = 0.0501]
Data/restraints/parameters	14863/0/768	17651/0/877
Goodness-of-fit on F^2	1.033	2.510
Final R indexes [I>=2σ (I)]	R_1 = 0.0281, wR_2 = 0.0746	R_1 = 0.1293, wR_2 = 0.3437
Final R indexes [all data]	R_1 = 0.0302, wR_2 = 0.0759	R_1 = 0.1431, wR_2 = 0.3493
Largest diff. peak/hole / e $Å^{-3}$	0.34/-0.47	2.37/-2.68

2. GPC traces of the polyethylenes

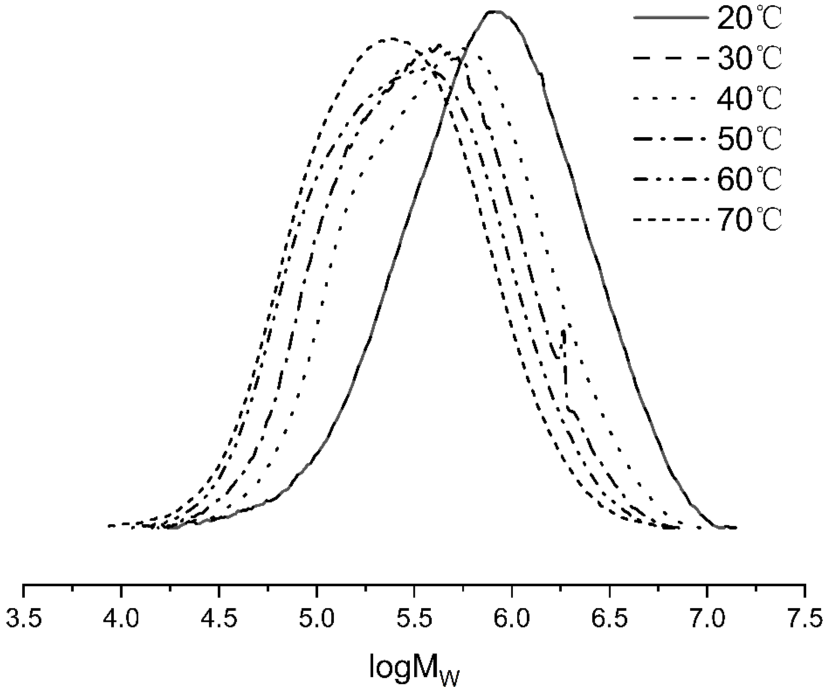

Figure S1 Molecular weight (log M_w) of the polyethylene produced using **Ni2**/$EtAlCl_2$ as a function of the run temperature (runs 1-6, Table 3).

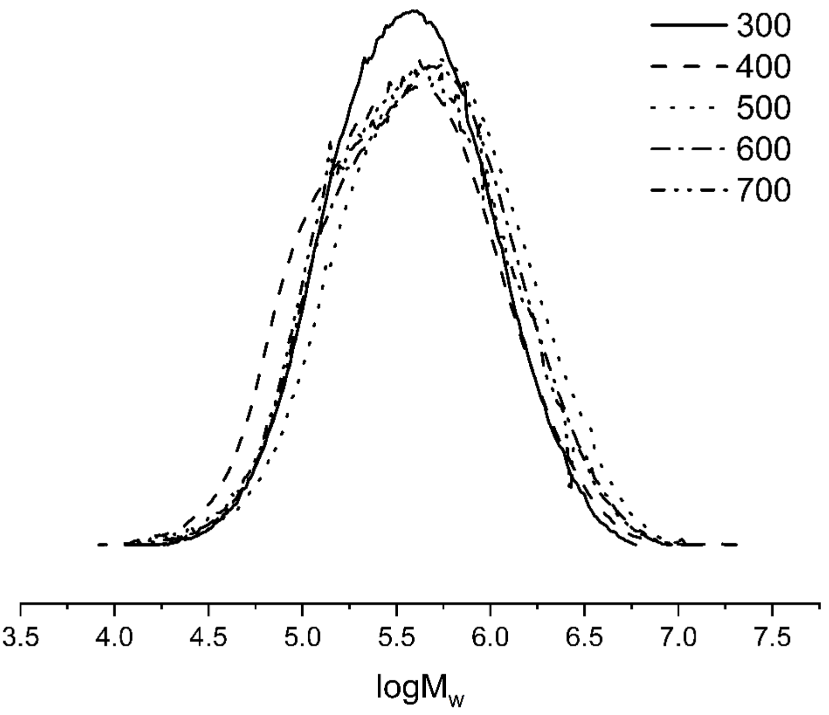

Figure S2 Molecular weight (log M_w) of the polyethylene produced using **Ni2**/$EtAlCl_2$ as a function of the Al:Ni molar ratio (runs 2 and 7-10, Table 3).

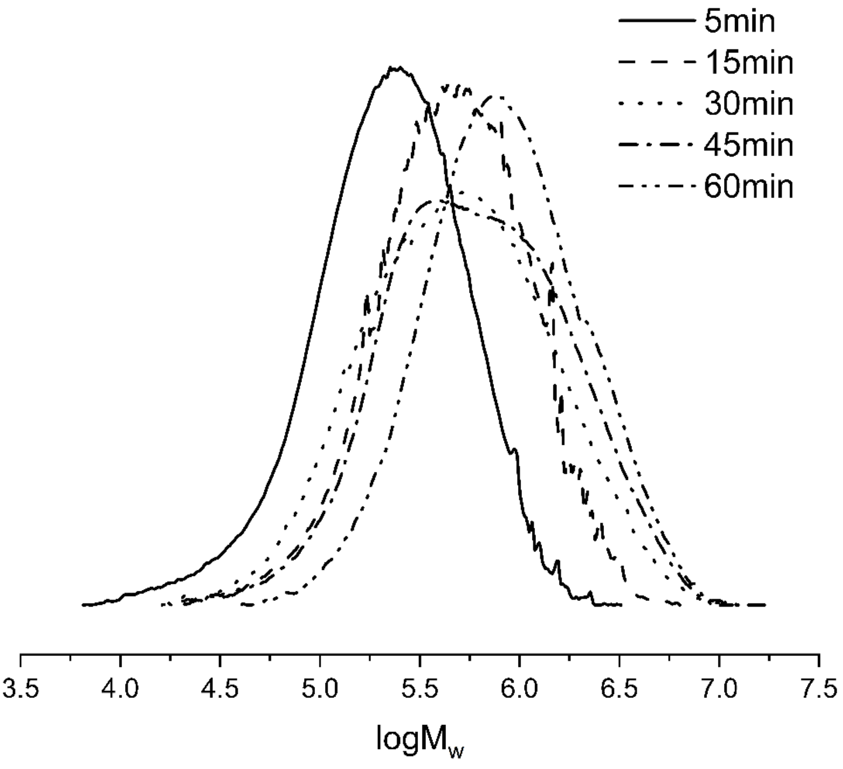

Figure S3 Molecular weight (log M_w) of the polyethylene produced using **Ni2**/$EtAlCl_2$ as a function of the run time (runs 2 and 11-14, Table 3).

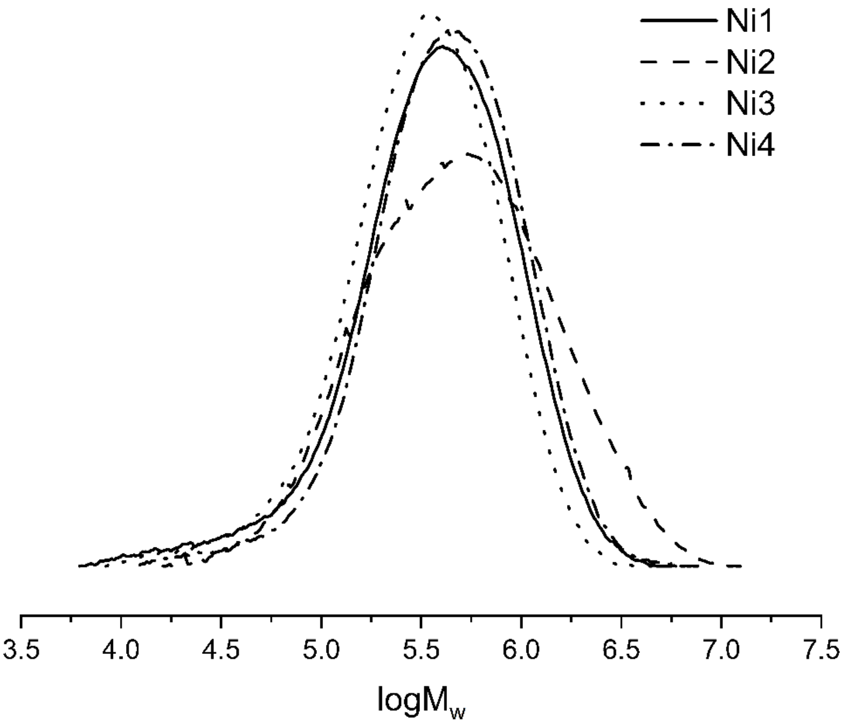

Figure S4 Molecular weight (log M_w) of the polyethylene produced using **Ni1**-**Ni4** under activation with $EtAlCl_2$ (runs 2 and 17-19, Table 3).

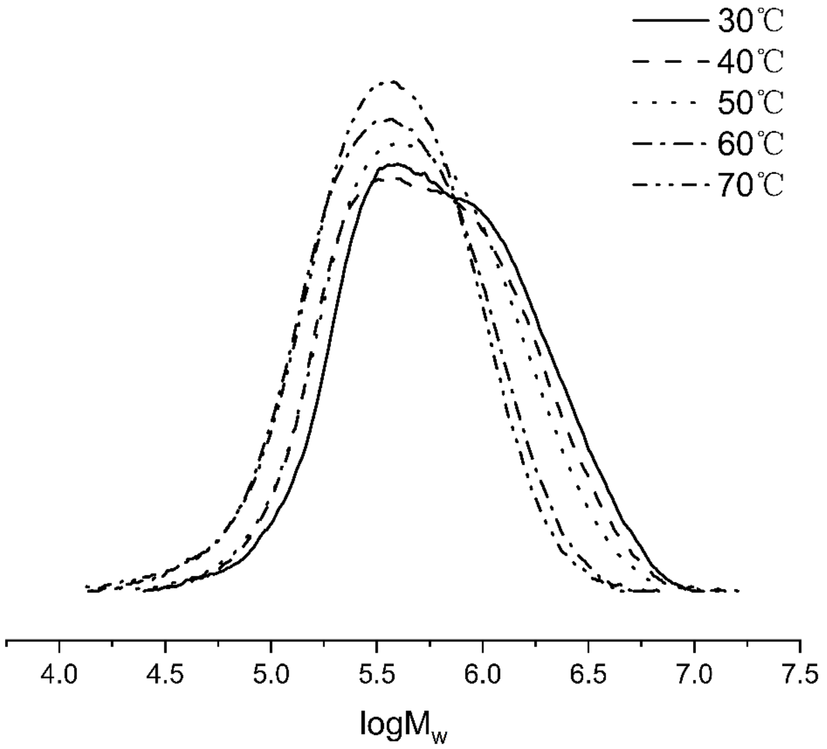

Figure S5 Molecular weight (log M_w) of the polyethylene produced using **Ni2**/Et_2AlCl as a function of the run temperature (runs 1-5, Table 4).

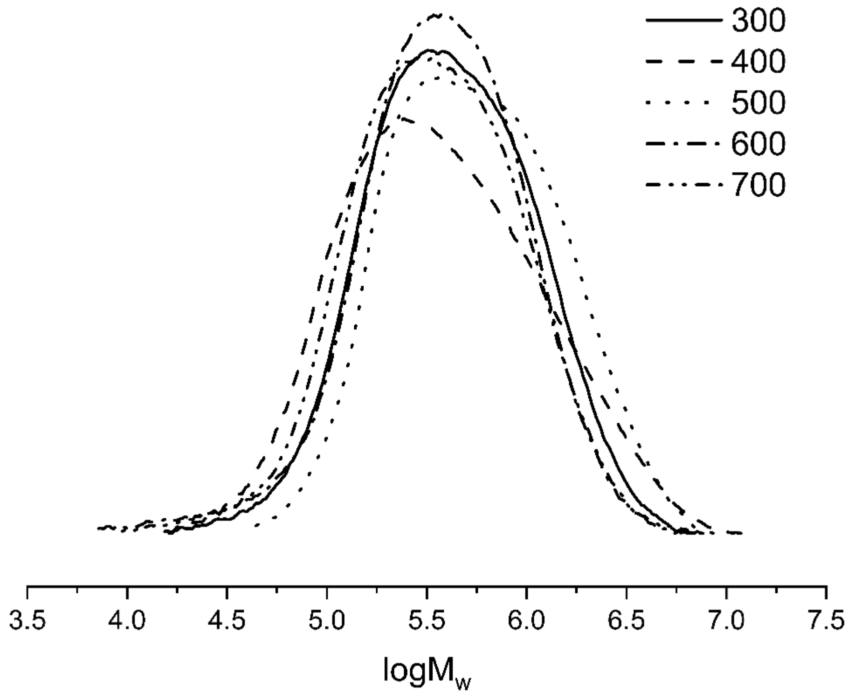

Figure S6 Molecular weight (log M_w) of the polyethylene produced using **Ni2**/Et_2AlCl as a function of the Al:Ni molar ratio (runs 2 and 6-9, Table 4).

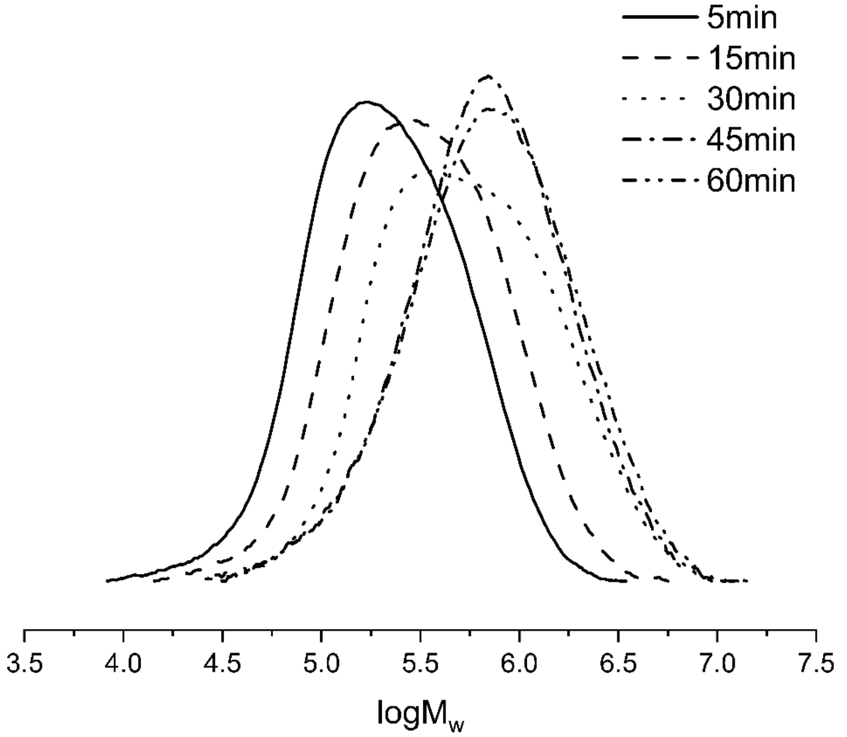

Figure S7 Molecular weight (log M_w) of the polyethylene produced using **Ni2**/Et_2AlCl as a function of the run time (runs 2 and 10-13, Table 4).

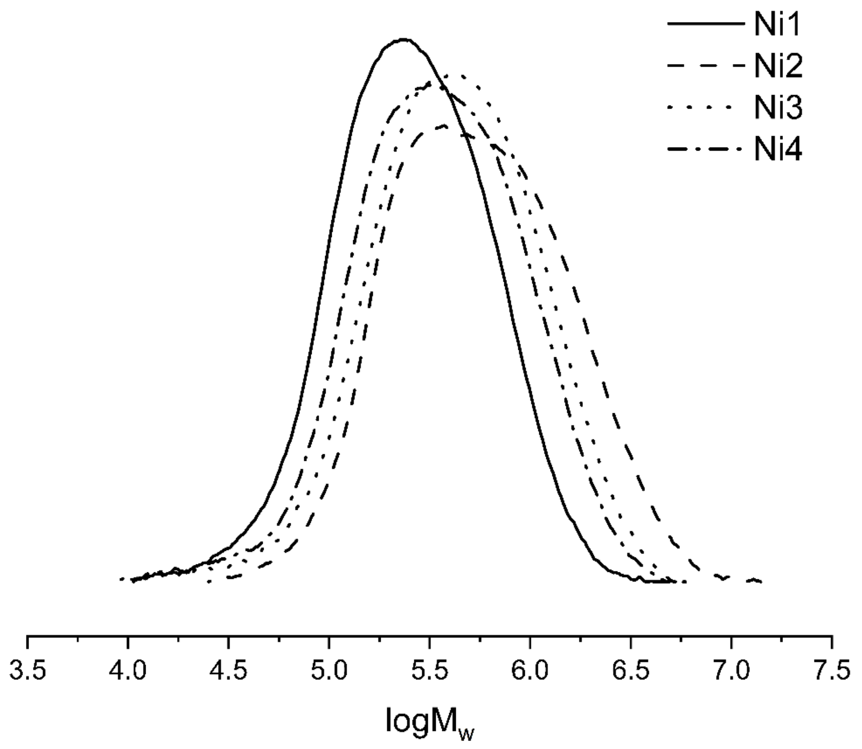

Figure S8 Molecular weight (log M_w) of the polyethylene produced using **Ni1**-**Ni4** under activation with Et_2AlCl (runs 2 and 16-18, Table 4).

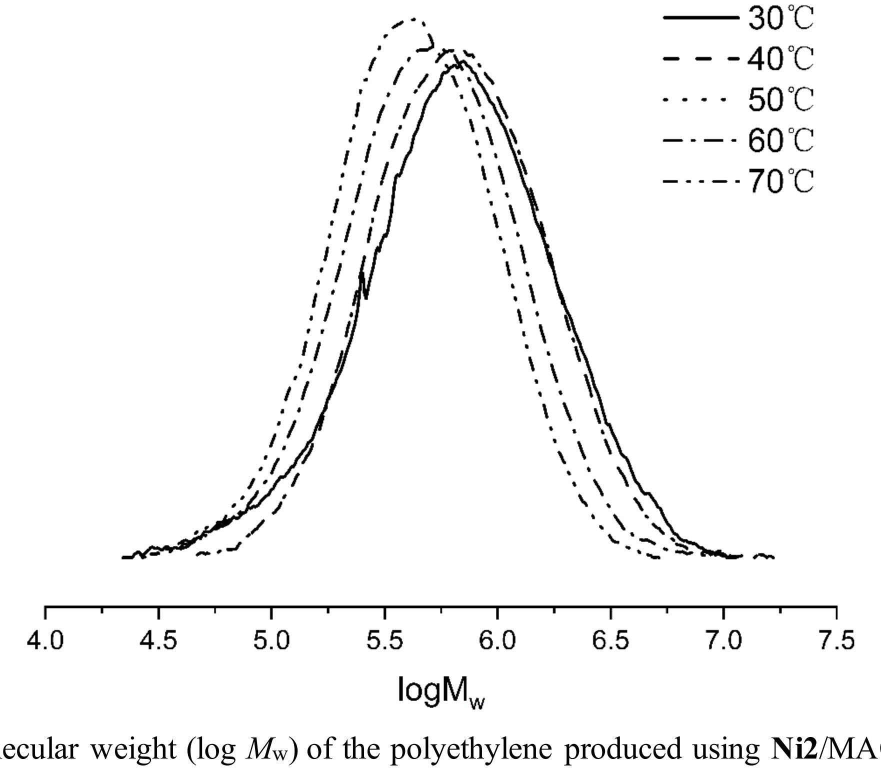

Figure S9 Molecular weight (log M_w) of the polyethylene produced using **Ni2**/MAO as a function of the run temperature (runs 1-5, Table 4).

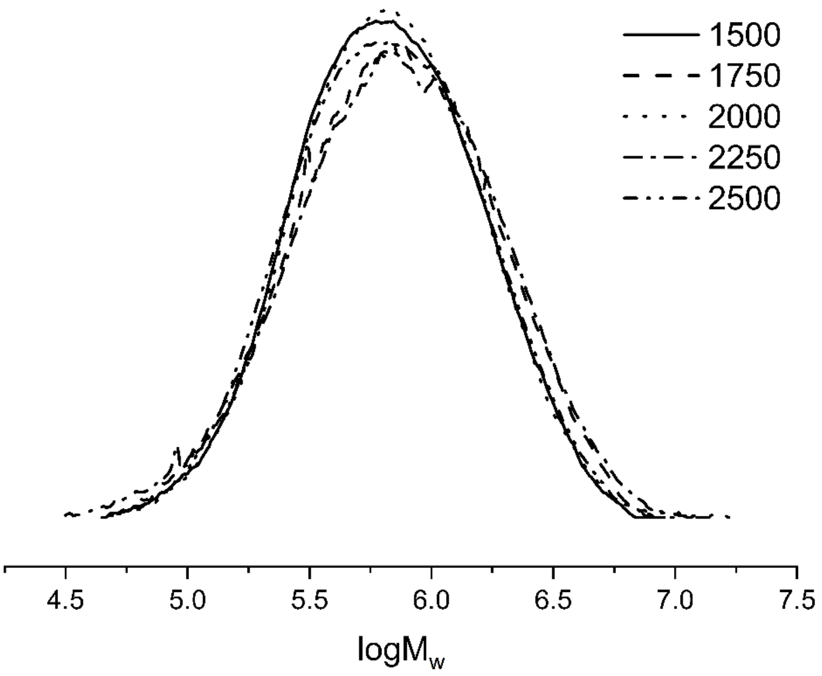

Figure S10 Molecular weight (log M_w) of the polyethylene produced using **Ni2**/MAO as a function of the Al:Ni molar ratio (runs 2 and 6-9, Table 5).

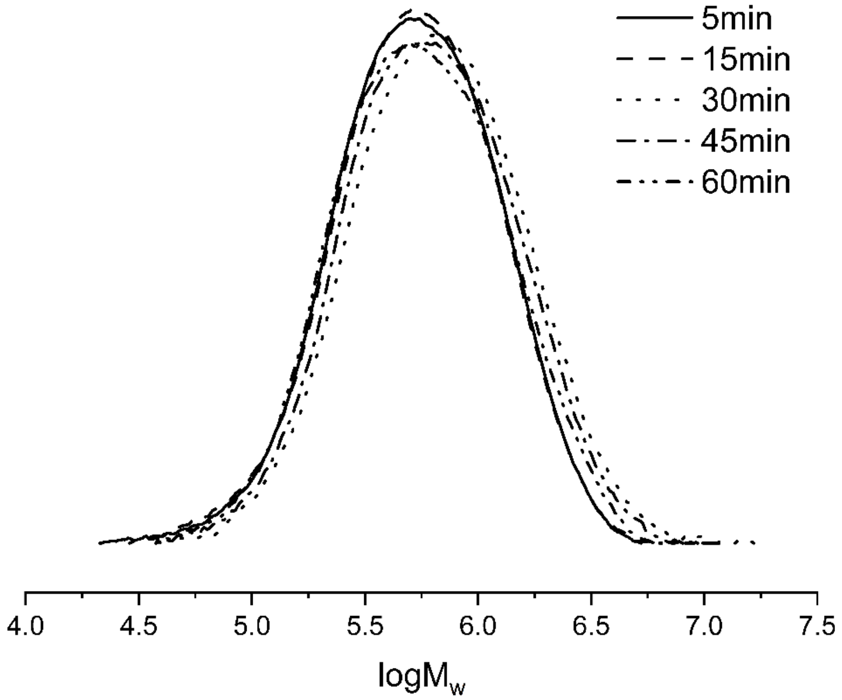

Figure S11 Molecular weight (log M_w) of the polyethylene produced **Ni2**/MAO as a function of the run time (runs 2 and 10-13, Table 5).

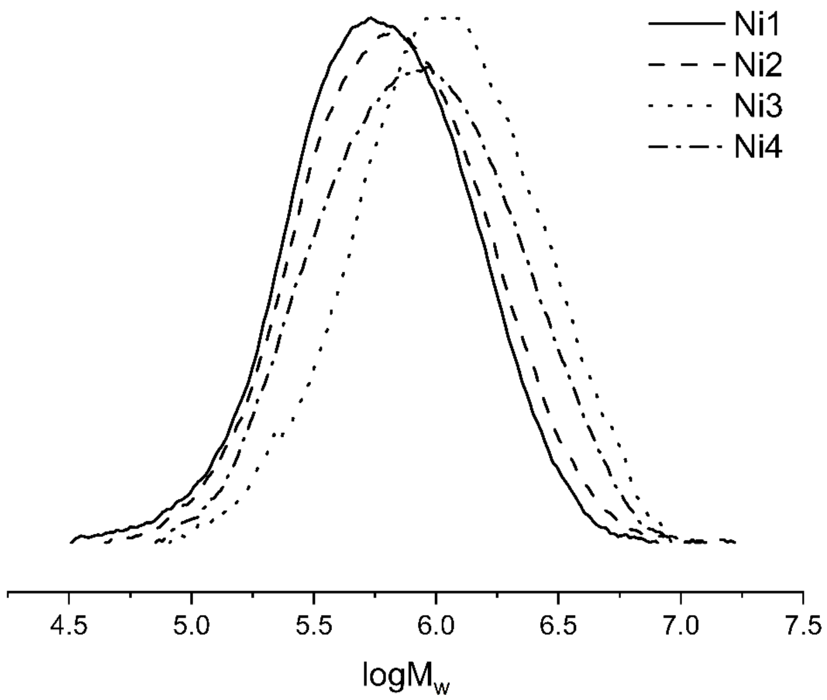

Figure S12 Molecular weight (log M_w) of the polyethylene produced using **Ni1**-**Ni4** under activation with MAO (runs 2 and 16-18, Table 5).

3. DSC thermograms of the polyethylenes

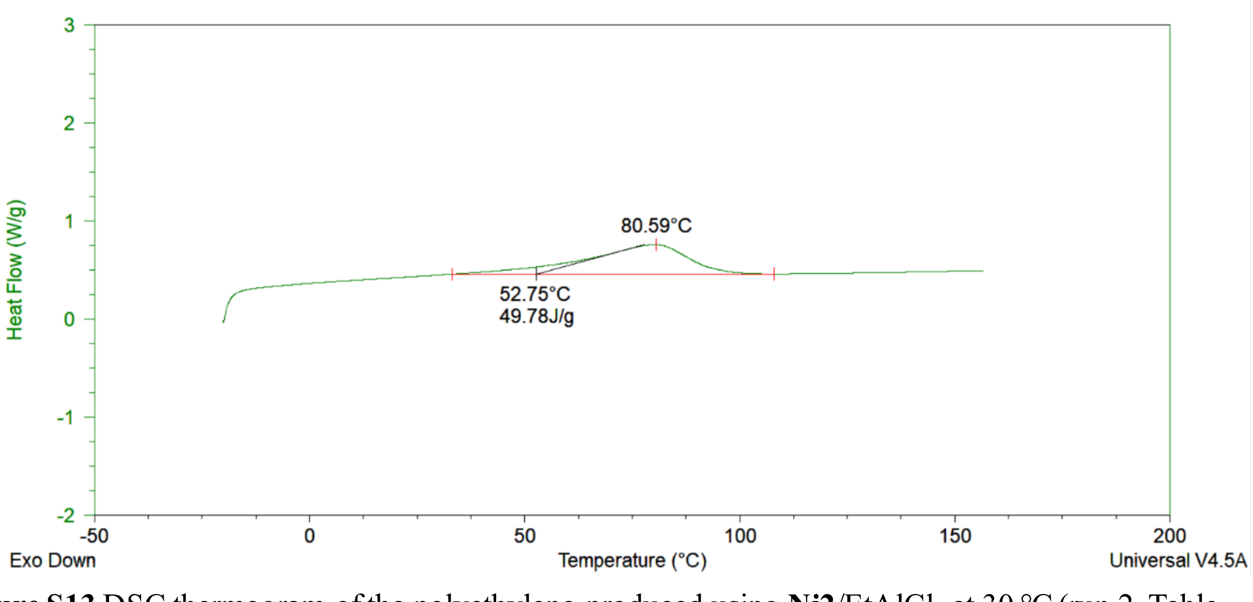

Figure S13 DSC thermogram of the polyethylene produced using **Ni2**/$EtAlCl_2$ at 30 °C (run 2, Table 3).

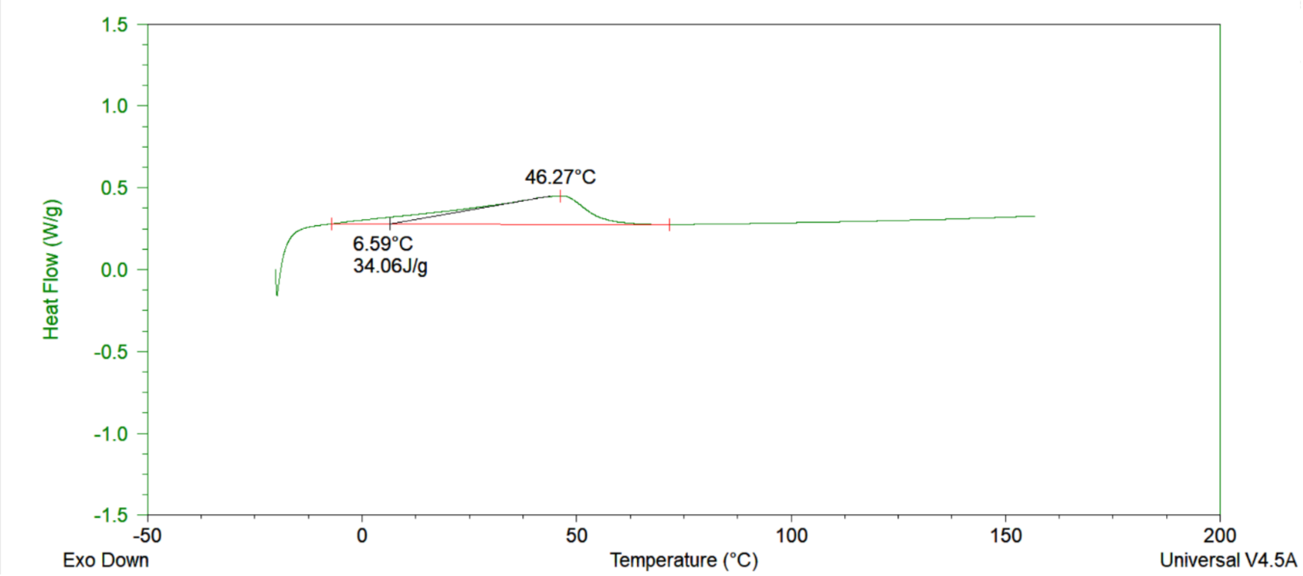

Figure S14 DSC thermogram of the polyethylene produced using **Ni2** and $EtAlCl_2$ at 60 °C (run 5, Table 3).

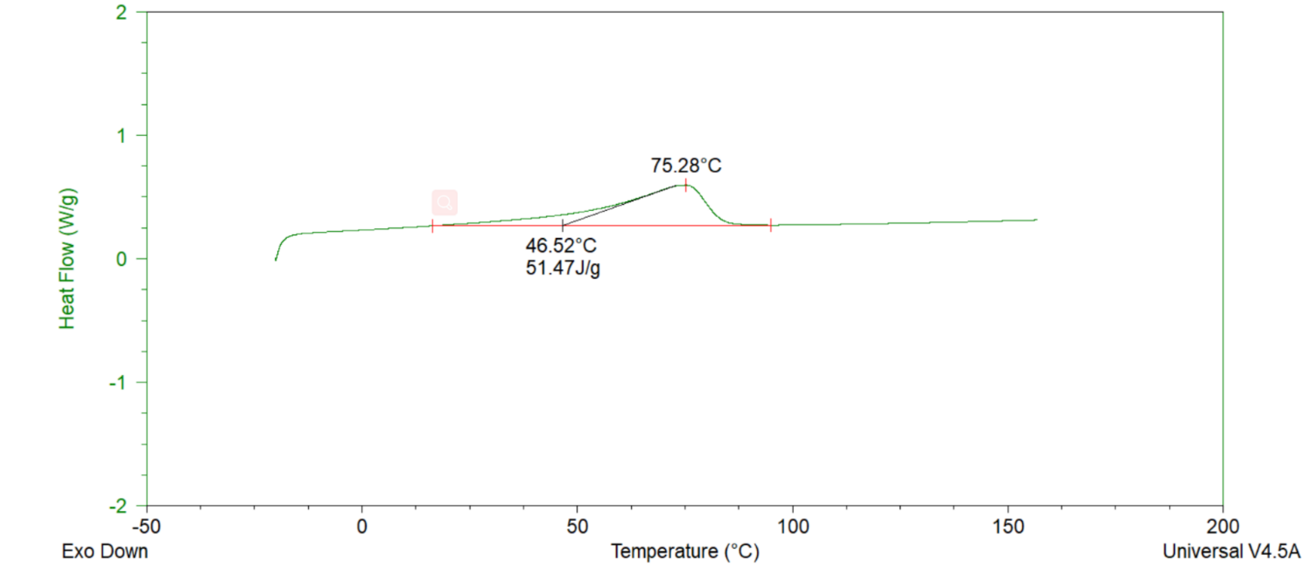

Figure S15 DSC thermogram of the polyethylene produced using **Ni2**/Et_2AlCl at 30 °C (run 1, Table 4).

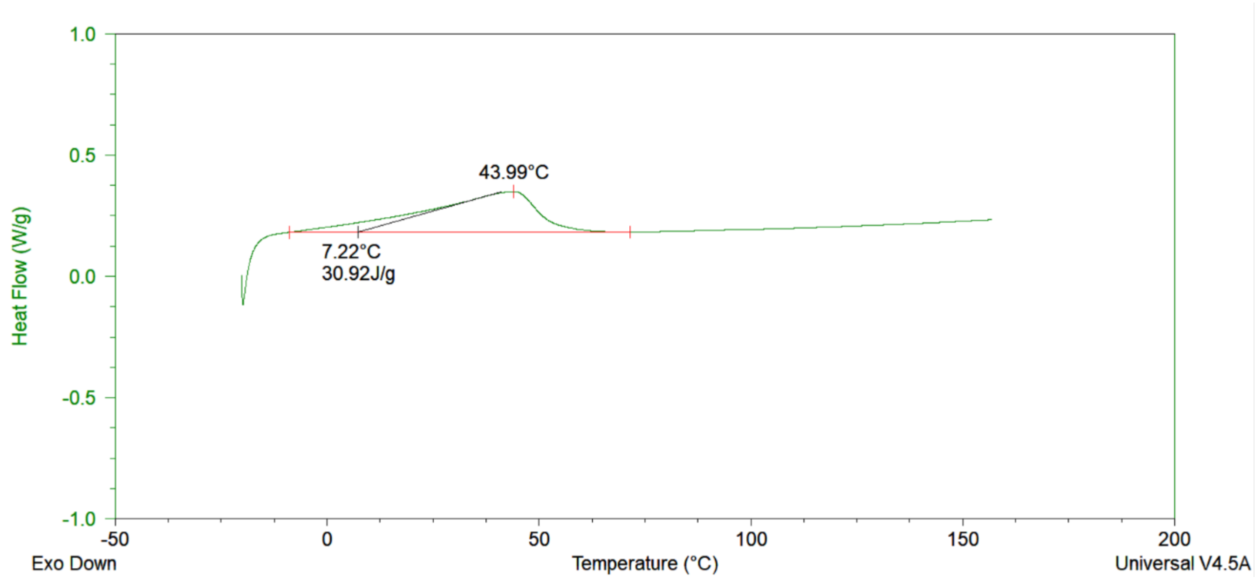

Figure S16 DSC thermogram of the polyethylene produced using **Ni2**/Et_2AlCl at 60 °C (run 4, Table 4).

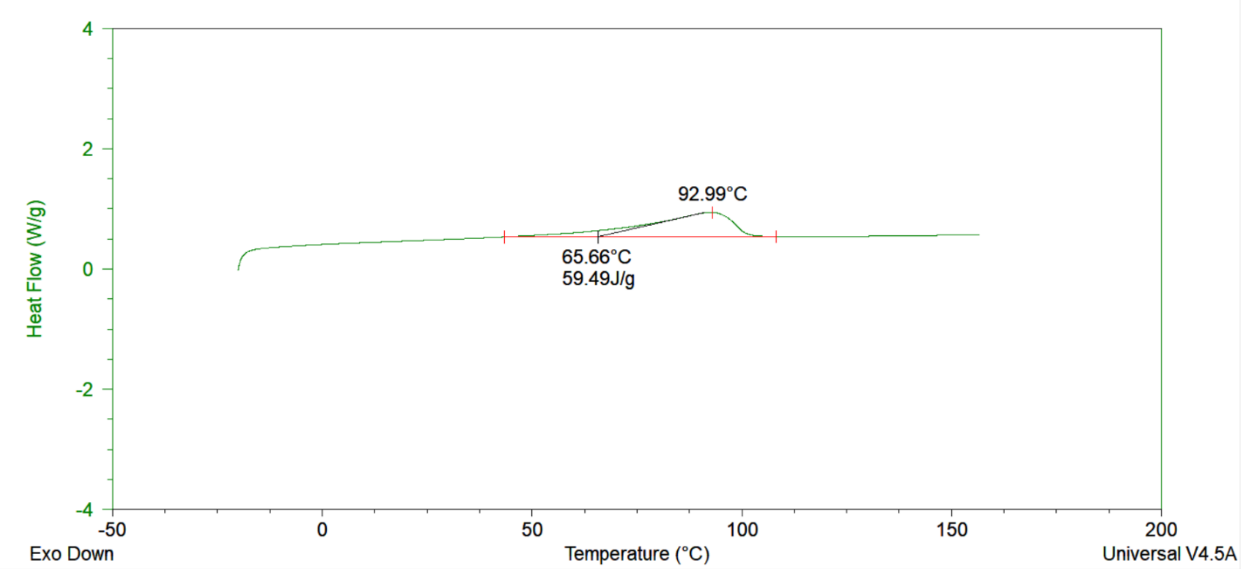

Figure S17 DSC thermogram of the polyethylene produced using **Ni2**/MAO at 30 °C (run 1, Table 5).

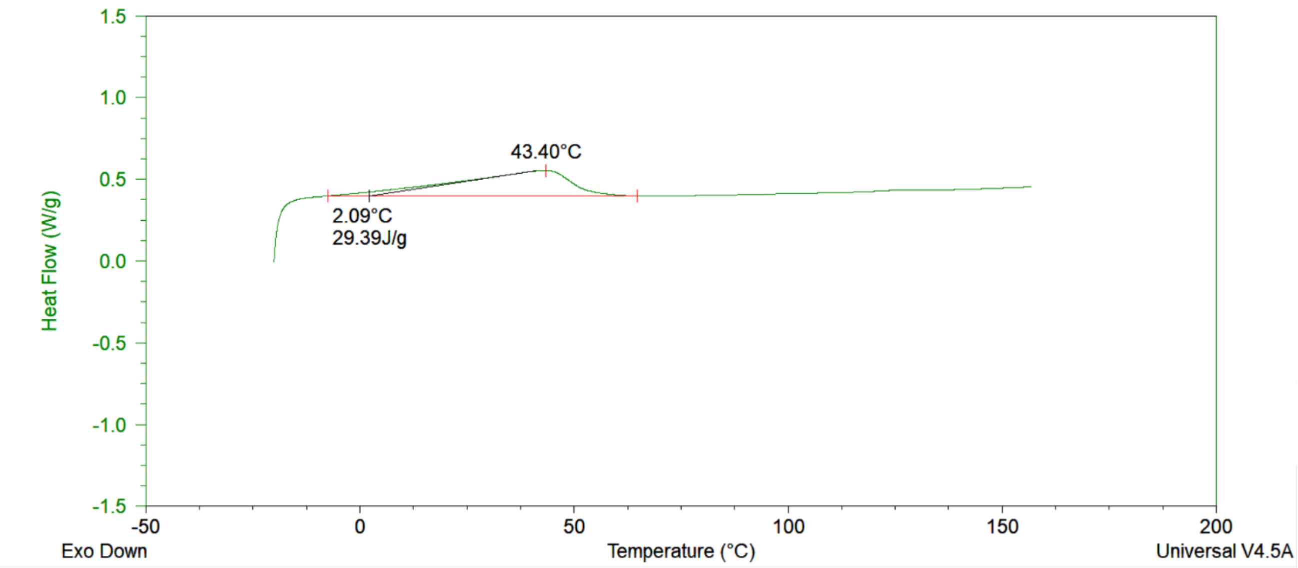

Figure S18 DSC thermogram of the polyethylene produced using **Ni2**/MAO at 60 °C (run 4, Table 5).
